# MOV10L1 Binds RNA G-Quadruplex in a Structure-Specific Manner and Resolves It More Efficiently Than MOV10

**DOI:** 10.1016/j.isci.2019.06.016

**Published:** 2019-06-15

**Authors:** Xia Zhang, Lina Yu, Shasha Ye, Jie Xie, Xingxu Huang, Ke Zheng, Bo Sun

**Affiliations:** 1 School of Life Science and Technology, ShanghaiTech University, Shanghai 201210, China; 2 State Key Laboratory of Reproductive Medicine, Nanjing Medical University, Nanjing, Jiangsu 211166, China; 3 Shanghai Institute of Biochemistry and Cell Biology, Chinese Academy of Sciences, Shanghai 200031, China; 4 University of Chinese Academy of Sciences, Beijing 100049, China

**Keywords:** Biological Sciences, Biochemistry, Molecular Biology

## Abstract

MOV10L1 and its paralog MOV10 are evolutionally conserved RNA helicases involved in distinct RNA regulatory pathways. The testis-specific MOV10L1 is essential for spermatogenesis and PIWI-interacting RNAs biogenesis, whereas MOV10 is ubiquitous and multifunctional. Although both proteins have been implied to correlate with RNA G-quadruplex (RG4) *in vivo*, their capabilities in binding and resolving RG4 and their respective biological significance remain unclear. Herein, we comprehensively characterize and compare the activities of these two helicases on various nucleic acid substrates *in vitro*, with a focus on RG4 structure. We find that both MOV10L1 and MOV10 are able to resolve RG4, with MOV10L1 being more efficient in that. In contrast to MOV10, MOV10L1 prefers to bind to a junction between single-stranded RNA and RG4, which is mediated by both its N and C termini. Furthermore, we show that RG4 unwinding by MOV10L1 facilitates the cleavage of this specific RNA structure by an endonuclease.

## Highlights

• Both MOV10L1 and MOV10 can resolve RG4 structure in an ATP-dependent manner
• MOV10L1 unwinds RG4 more efficiently than MOV10
• MOV10L1 preferentially binds to an ssRNA-RG4 junction
• RG4 unwinding by MOV10L1 facilitates its endonucleolytic cleavage

## Graphical Abstract

**Figure:**
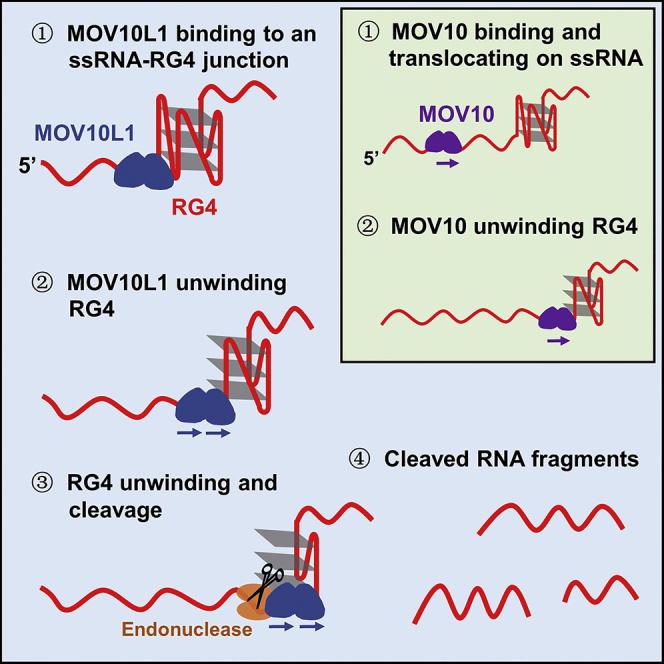

## Introduction

RNA helicases are ubiquitous motor enzymes that participate in nearly all aspects of RNA metabolism (Bourgeois et al., 2016, Jankowsky, 2011). Despite their importance, only a few eukaryotic RNA helicases have been functionally and kinetically characterized *in vitro* (Pyle, 2008). The mammalian paralogs Moloney leukemia virus 10 (MOV10) and MOV10-like 1 (MOV10L1) are RNA helicases belonging to superfamily 1 (SF1), and they exhibit 5′ to 3′ RNA helicase activity in unwinding RNA duplex *in vitro* (Gregersen et al., 2014, Vourekas et al., 2015). MOV10 is expressed in multiple tissues, and its diverse functions, such as retroelement inhibition and mRNA modulation, have been widely reported (Choi et al., 2018, Goodier et al., 2012, Gregersen et al., 2014). On the other hand, MOV10L1 is a testis-specific RNA helicase with a critical function restricted to male reproduction (Frost et al., 2010, Zheng and Wang, 2012, Zheng et al., 2010). The MOV10L1 helicase has been demonstrated to associate with PIWI proteins and control PIWI-interacting RNA (piRNA) biogenesis for retrotransposon silencing and protect the genome integrity of post-meiotic germ cells (Zheng and Wang, 2012, Zheng et al., 2010). Its point mutations K778A in the ATP-binding motif and DE888AA in the ATP hydrolysis motif cause loss of function of MOV10L1 in piRNA biogenesis and male fertility (Fu et al., 2016, Vourekas et al., 2015). However, the molecular mechanisms and functions of MOV10L1 in piRNA biogenesis are still obscure, although studies on mammalian piRNAs have provided a framework as to how piRNAs are generated (Fu and Wang, 2014, Hirakata and Siomi, 2016, Ku and Lin, 2014, Weick and Miska, 2014). Primary precursor transcripts require at least two critical steps to generate piRNAs. A first step with endonucleolytic cleavages on the primary piRNA precursor generates piRNA precursor intermediate fragments (PPIFs), which are loaded onto PIWI proteins that stabilize their 5′ ends (Vourekas et al., 2012, Vourekas et al., 2015). This is followed by a second step with 3′ to 5′ exonucleolytic trimming by PNLDC1 (Ding et al., 2017, Nishida et al., 2018, Zhang et al., 2017). Although MOV10L1 was proposed to mediate the initial step in piRNA processing when it binds the primary precursor transcripts to facilitate their endonucleolytic cleavage (Vourekas et al., 2015), a deepened mechanistic understanding of its molecular function as a helicase is crucial in piRNA biogenesis.

An intriguing feature shared by MOV10 and MOV10L1 from cross-linking and immunoprecipitation (CLIP)-seq analyses is that its preferable binding sequences harbor clusters of guanine (G) residues (Kenny et al., 2014, Vourekas et al., 2015). Their difference lies in the fact that MOV10L1-bound piRNA precursor transcripts that originate from genomic piRNA clusters are more enriched in G resides compared with those from other areas like MOV10-bound mRNAs (Vourekas et al., 2015). It has been long known that G-rich sequences in DNA and RNA have a propensity to fold into stable secondary structures termed G-quadruplexes (G4s), which are based on the stacking of several G-quartets with each layer consisting of four guanine bases held together by Hoogsteen-type hydrogen bonding (Bochman et al., 2012, Millevoi et al., 2012). Increasing evidence indicates that intramolecular RNA G-quadruplex (RG4) motifs are biologically relevant structures, and their occurrence can play vital roles in many key cellular functions, including telomere homeostasis, gene expression, and pre-mRNA processing (Agarwala et al., 2015, Bugaut and Balasubramanian, 2012, Cammas and Millevoi, 2017, Fay et al., 2017, Millevoi et al., 2012, Rhodes and Lipps, 2015, Simone et al., 2015). Even though several helicases and nucleases have been shown to remove DNA G-quadruplex (DG4) structure and regulate cellular processes (Baran et al., 1997, Chen et al., 2018, Harrington et al., 1997, Mendoza et al., 2016, Sauer and Paeschke, 2017, Sun et al., 1998, Sun et al., 1999), only a few RNA helicases, so far, have been reported to be capable of unwinding RG4 structures *in vitro* (Booy et al., 2012, Chakraborty and Grosse, 2011, Creacy et al., 2008, Gao et al., 2019, McRae et al., 2017, Ribeiro de Almeida et al., 2018). One of the reasons is that RG4 is a thermodynamically stable structure compared with other forms of RNA, thereby in requirement of specialized RNA helicase to resolve it (Hardin et al., 2000). The abundance of RG4s located within piRNA precursor along with an intimate coupling of piRNA precursor processing with RG4 raises the possibility that RG4 may exist as a structural mediator for the endonucleolytic cleavage of piRNA precursors, and MOV10L1 may take responsibility for resolving RG4 to facilitate the cleavage. However, whether and how the MOV10L1 helicase resolves the *bona fide* RG4 structures is unknown. In addition, in the unified model of PIWI-guided phased piRNA biogenesis, a question also remains how such a phasing process gets smoothly through a primary piRNA precursor bearing multiple RG4 obstacles (Gainetdinov et al., 2018, Vourekas and Mourelatos, 2018).

In this study, we employed robust biochemical assays to test the capability of MOV10L1 and MOV10 in binding and unwinding of RG4 structure *in vitro*. We found that even though both of them can unwind RG4, there are a few striking differences between them. MOV10L1 could take advantage of its unique features in RG4 binding and unwinding to mediate piRNA biogenesis. Last, a proof-of-concept assay with MOV10L1 and an endonuclease supports a model that they might cooperatively unwind and cleave RG4 structures in the initial step of piRNA biogenesis.

## Results

### MOV10L1 Requires ATP and 5′ ssRNA Tail to Resolve RG4 *In Vitro*

Helicase transduces the energy derived from NTP hydrolysis to unwind the duplex form of RNA and DNA (Singleton et al., 2007). First, we aimed to identify the optimal NTP as well as Mg^2+^ concentration for the unwinding activity of purified full-length MOV10L1 protein *in vitro* (Figure S1). Previous studies showed that the MOV10L1 helicase can unwind 5′-tailed short duplex RNA with a directionality of 5′ to 3′ (Vourekas et al., 2015). Thus, we designed a duplex RNA bearing an 18-nt 5′ overhang (referred to as 5′-tailed duplex substrate) to measure the helicase activity of MOV10L1 (Table S1 and Figure S2A). Comparison of the fraction of unwound substrate revealed most efficient unwinding in the presence of ATP and slightly lower but comparable unwinding efficiency in the presence of CTP or UTP, whereas GTP and all dNTPs supported very little unwinding (Figure S2B). Using similar method, we further tested the effect of Mg^2+^ concentration on the unwinding activity of MOV10L1 on this substrate. The unwinding fraction did not show a significant change in the range of 0.2 mM–2.5 mM Mg^2+^ (Figure S2C). In light of these results, we chose to use 2 mM ATP with 2 mM Mg^2+^ as the power source in the rest of our unwinding experiments.

**Figure 1:**
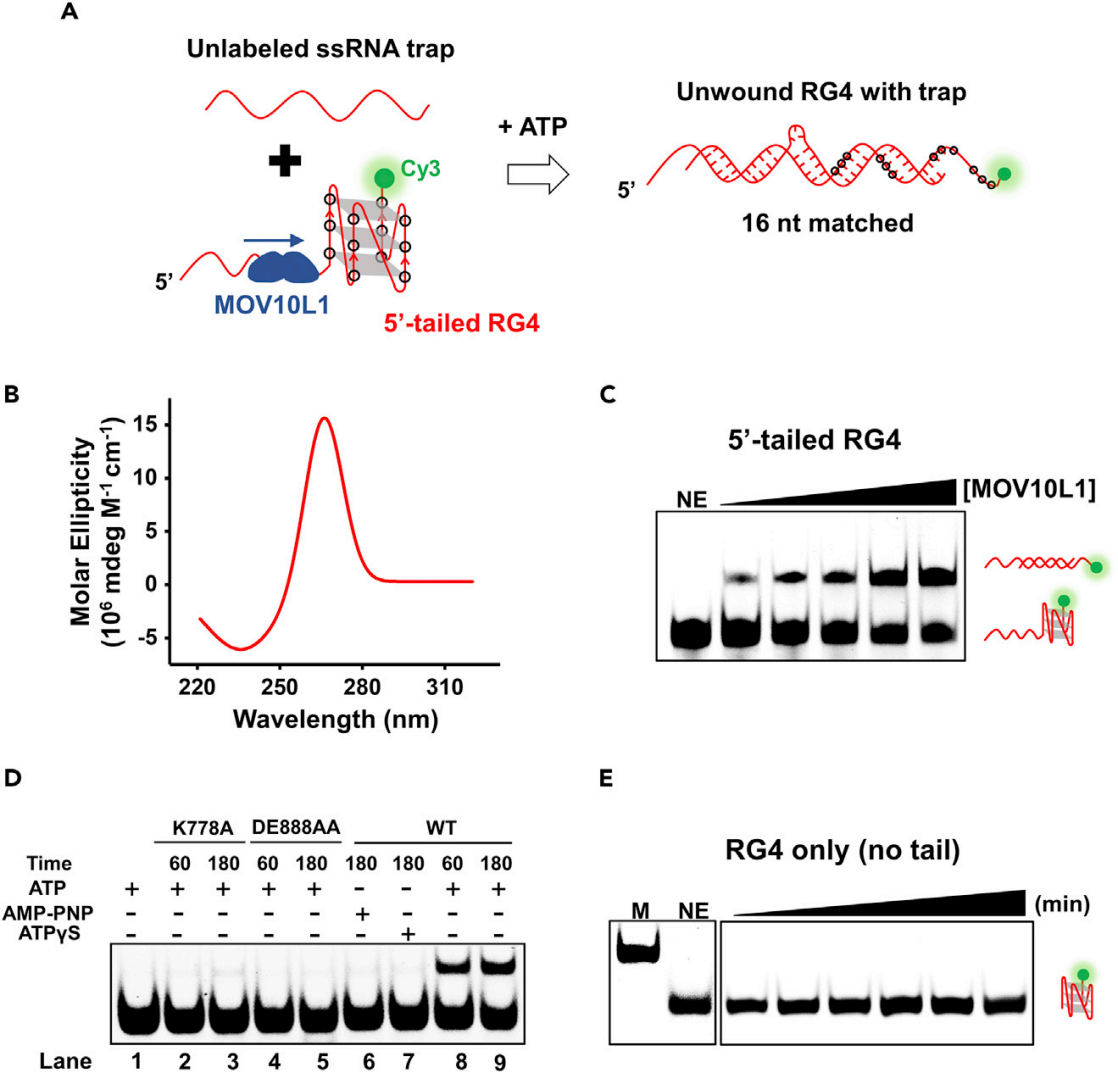
RG4 Unwinding by the MOV10L1 Helicase (A) Schematic diagram demonstrating the principle of the assay. 5′-tailed RG4 with cy3 fluorescence (green) labeled on the 3′ terminal is unable to base pair with the unlabeled trap RNA while folded into an RG4 structure. Base pairing occurs only if the RG4 is unwound in the presence of helicase and ATP. (B) CD spectrum of the RG4 substrate. (C) A representative image of MOV10L1-mediated 5′-tailed RG4-unwinding reactions with increasing protein concentration (0, 2, 4, 10, 20, 40 ng) at 37°C for 60 min. (D) 5′-tailed RG4 unwinding with mutant MOV10L1 (K778A or DE888AA) in the presence of ATP for the indicated period of times at 37°C. Wild-type (WT) MOV10L1-mediated RG4-unwinding reactions in the presence of ATP, AMP-PNP, or ATPγS were shown as well. (E) Representative gels of MOV10L1-mediated unwinding reactions with RG4-only substrate (no tails) with increasing time (0, 10, 30, 60, 120, 180 min) at 37°C in the presence of 10-fold ssRNA trap. M denotes marker prepared by replacing 100 KCl with 100 mM LiCl in the RG4 formation buffer. NE denotes no enzyme. All experiments were repeated three times.

As MOV10L1 was speculated to be involved in the unwinding of RG4 structure in piRNA precursor (Vourekas et al., 2015), we then examined that *in vitro*. To do so, we adopted a previously published approach with modifications to study the MOV10L1-mediated conversion of an intramolecular RG4 into a double-stranded (ds) RNA (Booy et al., 2015). Briefly, a 5′-tailed (16 nt) RG4 (referred to as 5′-tailed RG4 substrate) was formed by using a cy3-labeled RNA oligonucleotide (Figure 1A and Table S1, Transparent Methods). The circular dichroism (CD) spectrum of the sample showed a positive peak at 265 nm and a negative peak at 236 nm, which is the characteristic CD signature of an RNA quadruplex structure (Figure 1B) (Tang and Shafer, 2006), indicating that this substrate was properly folded into RG4 structure. MOV10L1 was pre-incubated with this substrate in the presence of Mg^2+^ and the unfolding was initiated by adding ATP. An unlabeled 25-nt single-stranded RNA (ssRNA) trap with 16 inconsecutive nucleotides complementary to the RG4 sequence was introduced along with ATP to prevent the unfolded RNA substrate from refolding (Figure 1A). As the unfolded RG4 substrate partially hybridized with the ssRNA trap, the resulting partial duplex is expected to show reduced mobility on a non-denaturing polyacrylamide gel by which the MOV10L1-mediated unwinding of RG4 can be monitored. Control experiments verified that this RG4 substrate could not be spontaneously unwound or converted to dsRNA in the presence of even 10 folds of the ssRNA trap in our experimental condition (Figure 1C). Unwinding assays indicated that MOV10L1 was indeed capable of resolving RG4 structure. The ability of MOV10L1 to unwind RG4 was dependent upon protein concentration (Figure 1C).

To find out whether the observed RG4 unwinding was caused by MOV10L1's helicase activity instead of other mechanisms, we sought to determine whether this activity depends on certain parameters that are compatible with a helicase mechanism. First, we tested the requirement of ATP hydrolysis as an energy source for RG4 unwinding. We performed a similar experiment in which the substrate was incubated with the MOV10L1 helicase in the presence of either ATP or its non-hydrolyzable analog, adenylyl-imidodiphosphate (AMP-PNP) or ATPγS. We found that RG4 was unwound and converted to dsRNA in the presence of ATP in this condition (Figure 1D, Lanes 8 and 9). In contrast, no obvious unwinding products were observed in the presence of AMP-PNP or ATPγS (Figure 1D, Lanes 6 and 7). To further confirm the unwinding activity detected was truly dependent on MOV10L1 instead of contaminations, we used point mutants of MOV10L1 in either its ATP-binding motif, K778A, or in its ATP hydrolysis motif, DE888AA. These two mutants have proved to be catalytically inactive (Fu et al., 2016, Vourekas et al., 2015). We observed that neither of these mutants exhibited RG4 unwinding activity in the presence of ATP (Figure 1D). These results confirm that ATP hydrolysis is required for MOV10L1 unwinding RG4.

Besides ATP, unwinding of the RNA duplex structure by MOV10L1 requires the substrate bearing a 5′ ssRNA tail (Vourekas et al., 2015). We then examined the necessity of the tail for MOV10L1 in RG4 unwinding. To do so, we designed a substrate containing RG4 sequence only (referred to as RG4 only substrate). A similar RG4-unwinding experiment was conducted as previously described with this substrate. In stark contrast with the 5′-tailed RG4 substrate, we found that the RG4-only substrate could not be unwound by MOV10L1, indicating the necessity of the 5′ ssRNA tail in MOV10L1-mediated RG4 unwinding (Figure 1E). Overall, we conclude that the MOV10L1 helicase can unwind RG4 structure in the presence of ATP and an ssRNA tail.

### MOV10L1 Unwinds RG4 More Efficiently Than MOV10

**Figure 2:**
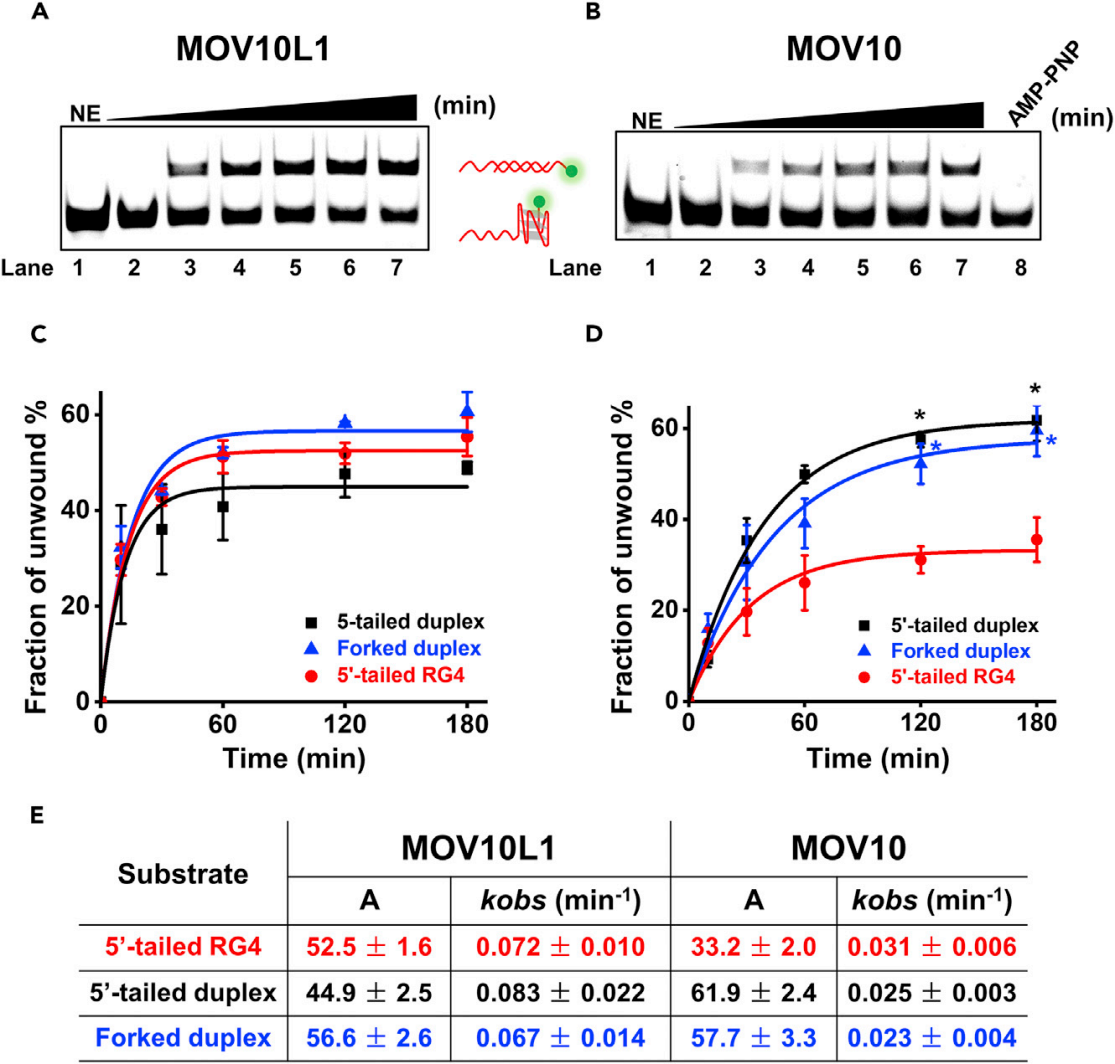
Unwinding Kinetics of MOV10L1 and MOV10 on Various RNA Substrates (A and B) Representative gels of 5′-tailed RG4 unwinding by the MOV10L1 helicase (A) and the MOV10 helicase (B) with increasing time (0, 10, 30, 60, 120, 180 min) at 37°C. (C and D) Quantitative analyses of MOV10L1 (C) and MOV10 (D) unwinding of 5′-tailed RG4, 5′-tailed RNA duplex, and forked duplex. Data shown are the means ± SD of three independent experiments. Values were compared with RG4 unwinding using Student's t test. *p < 0.05. (E) The values for the amplitudes (*A*) and rate constants (*k*) are tabulated.

Next, we sought to characterize RG4-unwinding kinetics by MOV10L1 and compared its unwinding efficiency with those of other RNA substrates or its paralog MOV10. We first conducted the RG4-unwinding assay in the presence of MOV10L1 as a function of time. As expected, the fraction of unwound RG4 substrate was increased over time (Figure 2A). For comparison, we also performed a series of unwinding experiments with 5′-tailed RNA duplex as well as forked RNA duplex in the presence of MOV10L1 (Figures S3A and S3B). Intriguingly, the fraction of unwound products with RG4 substrate was relatively higher than that with 5′-tailed duplex substrate, and comparable to that with forked RNA duplex substrate, although the reaction rates were slow for all three substrates (Figures 2C and 2E).

As MOV10 shares sequence similarity, to some extent, with MOV10L1, we next interrogated whether MOV10 might also be able to resolve RG4 *in vitro*. To test that, we performed the RG4 unwinding assay with purified MOV10 protein (Figure S1A). We found that the MOV10 protein can also unwind RG4 and the fraction of unwound substrate was increased over time (Figure 2B, Lanes 2–7). Control experiments with AMP-PNP showed no unwound products, confirming that ATP was required for MOV10's RG4 unwinding (Figure 2B, Lane 8). In comparison, both the maximum RG4-unwinding amplitudes and rates of MOV10 were significantly lower than those with MOV10L1 (Figures 2D and 2E). These observations can be explained as MOV10's unwinding activity is relatively weak or MOV10L1 preferentially unwinds RG4. To testify these two hypotheses, we compared MOV10's unwinding activities on the 5′-tailed RNA duplex and the forked RNA duplex substrates with MOV10L1 (Figures S3C and S3D). The unwinding amplitudes of both proteins were comparable on these two substrates and were almost twice that of MOV10 with the RG4 substrate, although the reaction rates of MOV10 were always slower than those of MOV10L1 (Figures 2B–2D). Given that RG4 structure is more stable than the duplex structures (Hardin et al., 2000), the fact that MOV10L1 unwinds RG4 comparably with other duplex substrates and more efficiently than MOV10 suggests that MOV10L1, compared with MOV10, prefers to unwind RG4 structure.

### MOV10L1 Preferentially Binds to an ssRNA-RG4 Junction

**Figure 3:**
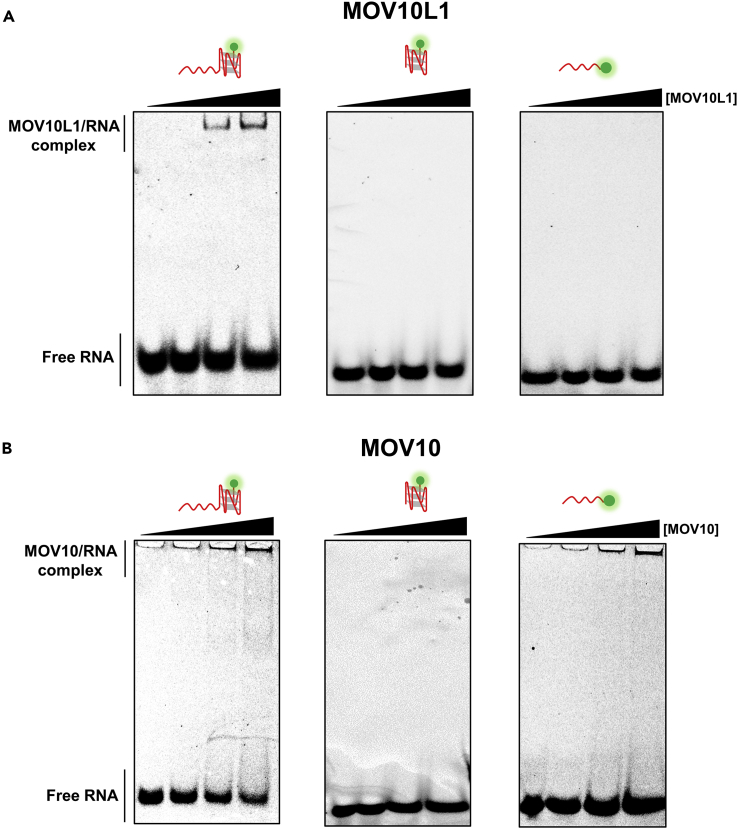
MOV10L1 Preferentially Binds to an ssRNA-RG4 Junction (A and B) Representative EMSA images of 2.5 nM of 5′-tailed RG4 substrate, RG4-only substrate, or 18-nt ssRNA substrate binding with MOV10L1 (A) and MOV10 helicase (B).

To gain further insight into the mechanisms of MOV10L1 in RG4 unwinding, we then investigated how 5′ ssRNA tail regulates MOV10L1 unwinding. As is the case for some helicases, they prefer to associate with ss nucleic acids first and unwind higher-order structures after translocating to the fork junction (Johnson et al., 2007). Alternatively, helicases may utilize their ability to recognize their substrates in a structure-specific manner, such as fork junction and D loop structures, before unwinding (Anand and Khan, 2004, Popuri et al., 2008). Thus the inability of MOV10L1 to unwind RG4 only substrate could be possibly attributed to the lack of either an ssRNA tail as a loading region or a specific junction formed by ssRNA and RG4. To testify these two hypotheses, we examined the binding affinity of MOV10L1 to various types of RNA substrates using an electrophoretic mobility shift assay (EMSA). We first incubated the 5′-tailed RG4 substrate with purified MOV10L1 in the presence of AMP-PNP. As expected, a band shift was observed upon gel electrophoresis as a result of MOV10L1/RG4 complex formation (Figure 3A). In contrast, the complex was not observed with the RG4-only substrate, suggesting that the inability of MOV10L1 to unwind this substrate is at least partially due to the weak binding of MOV10L1 to the template (Figure 3A). Surprisingly, MOVL10L1 also exhibited no binding affinity to an 18-nt ssRNA substrate either (Figure 3A). These results indicate that association of MOV10L1 with RNA substrates necessitates the existence of both the ssRNA and RG4 and strongly suggest that MOV10L1 might specifically bind at a junction between ssRNA and RG4 structure. Next, we repeated the same EMSA assays with the MOV10 protein and found that it exhibited similar binding behavior on RG4-only and 5′-tailed RG4 substrates with MOV10L1 (Figure 3B). However, surprisingly, in stark contrast to MOV10L1, MOV10/ssRNA complex was also detected (Figure 3B). These results proved that the binding preference of MOV10L1 on a fork might be quite unique and is not conserved in its family.

To further verify MOV10L1's preferential binding to a junction, we performed similar experiments with various RNA duplex substrates. As expected, MOV10L1/RNA complexes were observed with either the 5′-tailed or the forked RNA duplex substrate (Figure S4). However, no binding complex was detected with a blunt duplex RNA substrate (no ssRNA tail) (Figure S4). Clearly, an ssRNA tail is a necessity for MOV10L1 binding to RNA substrate.

Taken together, we conclude that MOV10L1 has a structure-specific binding manner, and the presence of an unpaired 5′ ssRNA region promotes its binding to the structured RNA substrate.

### Neither MOV10L1 or MOV10 Can Unwind DG4

**Figure 4:**
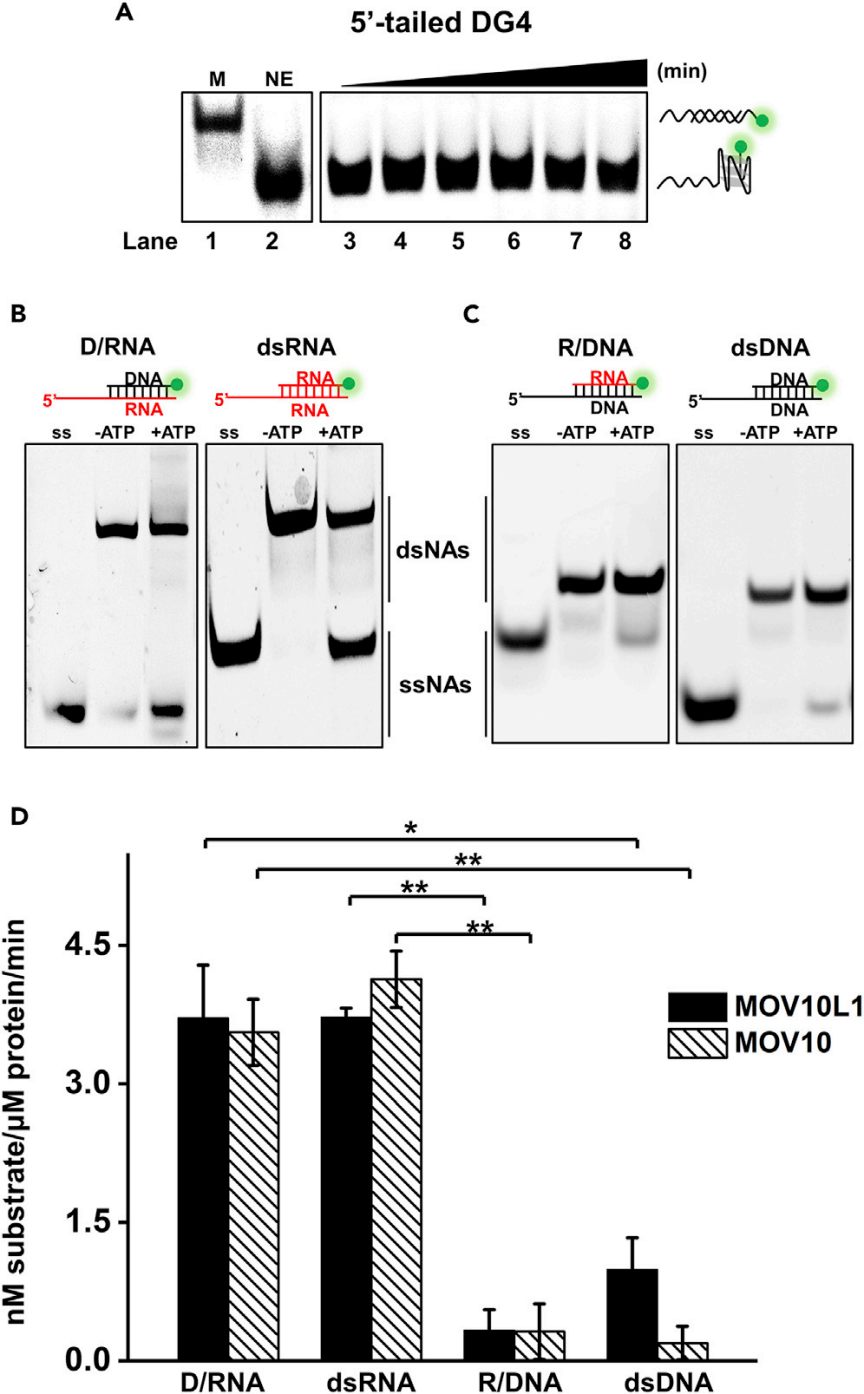
Unwinding Activities of MOV10L1 and MOV10 on Various Substrates (A) A representative gel of MOV10L1-mediated DG4-unwinding reactions at 0, 10, 30, 60, 120, and 180 min. Marker (denoted as M) was prepared as described in the Transparent Methods section. (B and C) MOV10L1 unwound different duplex substrates (D/RNA, dsRNA, R/DNA, and dsDNA, 10 nM each) with or without ATP at 37°C for 180 min. dsNAs and ssNAs denote double-stranded and single-stranded nucleic strands, respectively. (D) The quantitative analyses of gels in (B) and (C). Data shown are the means ± SD of three independent experiments. Values were compared using Student's t test. *p < 0.05, **p < 0.01.

Many helicases have been found to possess dual unwinding activities, acting on both DNA and RNA substrates. Thus far, there are seven helicases exhibiting RG4-unwinding activity and two of them have been identified to unwind DG4 structure as well (Booy et al., 2012, Chakraborty and Grosse, 2011, Gao et al., 2019). We then asked whether MOV10L1 can also work on DG4 substrate. To examine that, a cy3-labeled DG4 substrate bearing a 5′ ssDNA tail (referred to as 5′-tailed DG4 substrate) was used. This substrate was pre-incubated with MOV10L1, and unfolding assay was initiated by adding ATP and an unlabeled 25-nt ssDNA trap with 16 nt complementary to the DG4 sequence. Control experiments confirmed that this DG4 substrate could not be spontaneously resolved in the presence of the ssDNA trap (Figure 4A, Lane 2). However, we also did not observe obvious DG4-unwinding products in the presence of MOV10L1 and ATP (Figure 4A). One of the explanation for that could be MOV10L1 preferentially tracks along the backbone of ssRNA over ssDNA. To verify that, we designed a family of 5′-tailed dsDNA and RNA-DNA hybrid substrates (Figures 4B and 4C). The unwinding reactions were carried out as described previously. We first examined the unwinding activity of MOV10L1 on DNA-RNA hybrid substrate in which tailed strand is RNA (referred to as D/RNA). MOV10L1 exhibited nearly no unwinding activity in the absence of ATP but efficient unwinding activity on this template when ATP was present, and the fraction of unwound substrates was similar to the one with the dsRNA substrate (Figures 4B and 4D). Next, we examined the unwinding activities of MOV10L1 on dsDNA and RNA-DNA hybrid (referred to as R/DNA) substrates in which tailed strands are DNA. We found that, compared with substrates containing RNA tails, MOV10L1 displayed very weak unwinding activities on both substrates (Figures 4C and 4D). We also examined MOV10's unwinding activities with all these substrates, and similar results were recorded (Figures S5 and 4D). In summary, MOV10L1/MOV10 is able to displace short DNA and RNA strands, provided that the loading or tracking strand is ssRNA. Neither of them can resolve DG4, possibility due to their preference of ssRNA over ssDNA.

### Both the N and C Termini of MOV10L1 Are Required for RG4 Unwinding

**Figure 5:**
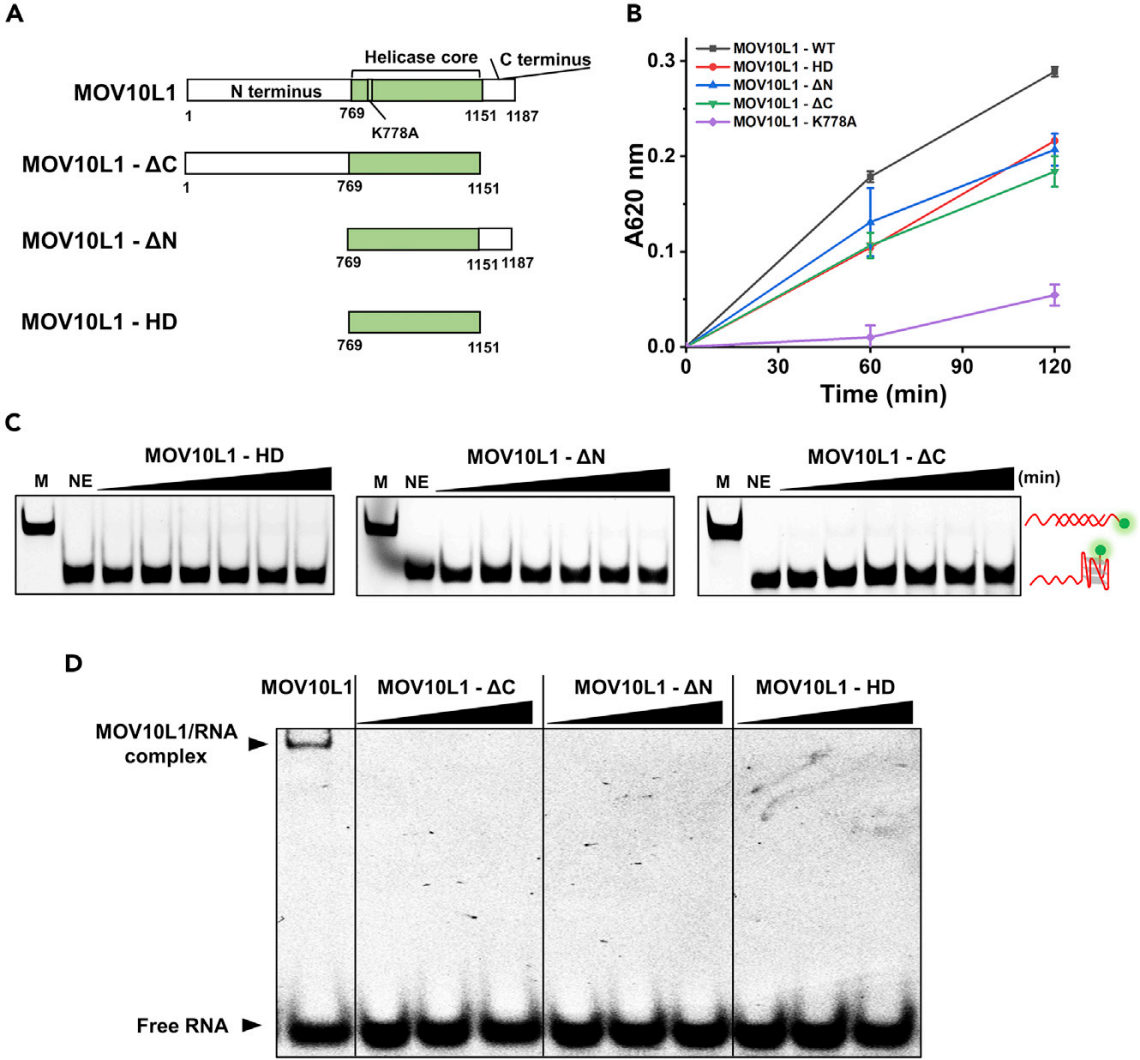
Both the C and N termini of MOV10L1 Are Required for RG4 Binding and Resolving (A) Schematic representation of the MOV10L1 full-length domain structure and mutant MOV10L1 proteins used in this study. An ATP-binding-deficient point was mutated to create mutant MOV10L1 (K778A). (B) The ATPase activity of the WT MOV10L1 and its mutants in the presence of ssRNA. Data shown are the means ± SD of three independent experiments. (C) Truncated MOV10L1 proteins (MOV10L1-HD, MOV10L1-ΔN, and MOV10L1-ΔC) unwound the 5′-tailed RG4 substrate with increasing time (0, 10, 30, 60, 120, 180 min) at 37°C. (D) A representative EMSA gel for truncated MOV10L1 proteins binding to a 5′-tailed RG4 substrate (2.5 nM) with increasing protein concentrations (0, 2, 10, 20 ng). All experiments were carried out three times.

It is widely known that besides the helicase core domain, the helicase function often needs to be activated through extra domains (Fiorini et al., 2013, Rudolph and Klostermeier, 2015). The N- and C-terminal domains of helicases often regulate their substrate specificity and cellular functions. To address whether the ancillary domains of MOV10L1 also regulate its RG4 unwinding, we purified the helicase core domain of MOV10L1 helicase (referred to as MOV10L1-HD) and examined its unwinding activities on the 5′-tailed RG4 substrate (Figure 5A and Table S2). Probing for the FLAG tag by western blot confirmed that all protein mutants were expressed at roughly equivalent levels (Figure S1B). Using a helicase-coupled ATPase assay, we confirmed that in contrast with K778A, the wild-type and all truncated MOV10L1 variants exhibited pronounced RG4-stimulated ATPase activity, confirming that the truncated MOV10L1 mutants were folded into active conformations (Figure 5B). We carried out similar RG4-unwinding experiments to that described above except with the MOV10L1-HD protein. We found that MOV10L1-HD exhibited no unwinding activity on this substrate at all (Figure 5C). This result indicates that either the N- or the C-terminal domain is necessary for MOV10L1 to resolve RG4. To test which domain is indispensable in RG4 unwinding, we further purified MOV10L1 missing either the N or C terminus (referred to as MOV10L1-ΔN and MOV10L1-ΔC, respectively, Figure 5A and Table S2) and examined their unwinding activities on RG4 structure. Remarkably, neither of them was able to resolve RG4 structure in our experimental condition, suggesting that both termini are indispensable for MOV10L1 unwinding RG4 (Figure 5C). To further confirm the above conclusions, we also examined the unwinding activities of these mutants on the 5′-tailed RNA duplex substrate. Consistently, we barely observed unwinding activities of these three mutants with this substrate (Figure S6).

The ancillary domains of helicase are often found to regulate helicase unwinding activities by mediating its association with substrates; we thus examined the binding affinities of these mutants to the 5′-tailed RG4 substrate using an EMSA approach similar to the one described above except with MOV10L1 truncated mutants. We found that none of these three mutants could effectively form complex with the substrate (Figure 5D). Taken together, we conclude that both the N- and C-terminal domains of MOV10L1 helicase are indispensable for its unwinding activity, and these two domains participate in regulating MOV10L1's association with RNA substrates.

### RG4 Unwinding by MOV10L1 Facilitates Its Endonucleolytic Cleavage

**Figure 6:**
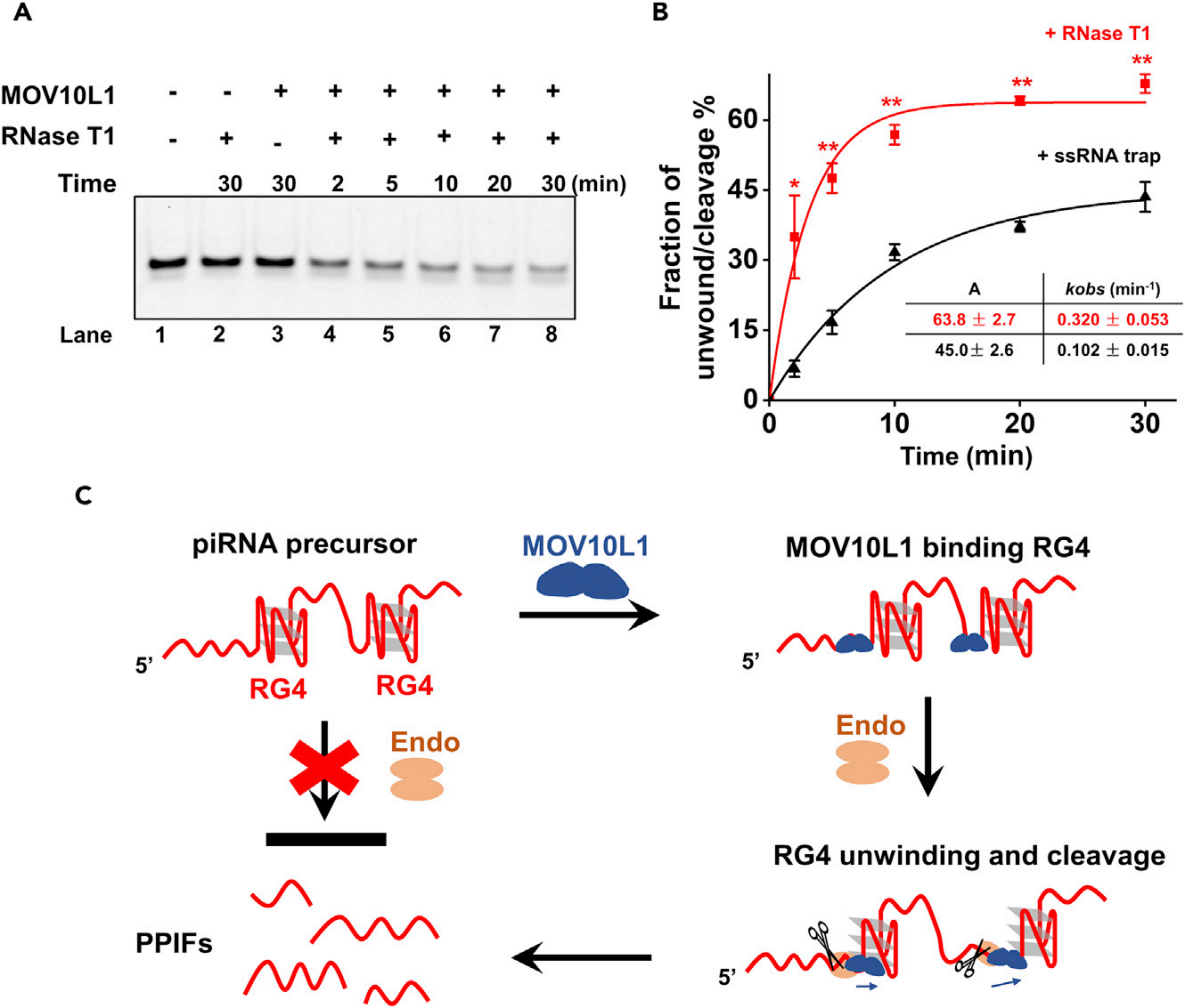
RG4 Unwinding and Cleavage by Endonuclease RNase T1 and MOV10L1 (A) MOV10L1 protein unwound 5′-tailed RG4 in the presence of 50 U of RNase T1 with increasing time (0, 2, 5, 10, 20, 30 min) at 37°C. This experiment was repeated three times. Reactions without MOV10L1 or RNase T1 acted as negative controls. (B) The quantitative analyses of MOV10L1-mediated RG4 unwinding in the presence of either the ssRNA trap or RNase T1. The values for the amplitudes (*A*) and rate constants (*k*) are tabulated in the panel inset. Data shown are the means ± SD of three independent experiments. Values were compared using Student's t test. *p < 0.05, **p < 0.01. (C) A model of MOV10L1's functions in piRNA biogenesis. RG4s exist in piRNA precursor, which prevents the endonucleolytic cleavage by the endonuclease (endo). MOV10L1 specifically binds to these structures bearing a 5′ RNA tail at the junctions. PPIFs are generated with the assistance of helicase unwinding and endonuclease cleavage activities. Dark blue arrows indicate helicase-unwinding direction, and scissors indicate the cleavage activity of the endonuclease.

Previous studies suggest that the MOV10L1 helicase might participate in piRNA biogenesis in a manner that it resolves RNA secondary structures in piRNA precursor to facilitate its endonucleolytic cleavage *in vivo* (Vourekas et al., 2015). To dissect MOV10L1's functions in piRNA biogenesis, we examined if the MOV10L1 helicase could facilitate the digestion of an RG4 structure and how its RG4-unwinding activity would be affected by the cleavage. As the exact endonuclease accounting for the 5′ end formation of piRNA intermediates is still under debate (Gainetdinov et al., 2018, Nishida et al., 2018), we chose to use RNase enzyme T1, which specifically cuts after guanines at the ssRNA region. We designed a 5′-tailed RG4 substrate with no guanine contained in the tail region (referred to as RG4-2, Table S1). This substrate was found to be resistant to RNase T1 cleavage once the RG4 structure was folded (Figure 6A, Lane 2). When both MOV10L1 and RNase T1 were present, the RG4 template was effectively digested over time in the absence of the ssRNA trap, suggesting that MOV10L1 indeed facilitates RNase T1 digestion of RG4 (Figure 6A, Lanes 4 - 8). This might occur in a manner that the MOV10L1 helicase resolves RG4 structure to expose guanine residues, allowing them for the cleavage. Moreover, compared with MOV10L1-mediated unwinding of this RG4-2 template in the presence of an ssRNA trap, both the amplitude and the unwinding rate of the unwinding or cleavage reactions were enhanced with the presence of RNase T1 (Figures 6B and S7). It is likely that RG4 unwinding by MOV10L1 is in competition with RG4 folding and that if immediately cleaved (in this case by RNase T1), unwinding is more effective.

## Discussion

High-throughput reverse transcription sequencing methods have identified over 12,000 RG4s in the transcriptome (Guo and Bartel, 2016, Kwok et al., 2016). Moreover, intermolecular RG4 structures are also found to exist in the tRNA fragment as a critical component of cellular stress response (Lyons et al., 2017). During the past decade, increasing evidence indicates that intramolecular G4 motifs are biologically relevant structures, and their occurrence can have negative or positive roles within the cell (Rhodes and Lipps, 2015). However, a recent study showed that endogenous RNA regions that have potential to form RG4s are globally unfolded *in vivo* in eukaryotic cells, indicating the existence of robust and effective machineries, such as helicases (Guo and Bartel, 2016). Thus far, only seven RNA helicases (DBP2, DED1, MSS166, DHX36, DDX21, DDX1, and DHX9) have been reported to unwind RG4 (Booy et al., 2012, Chakraborty and Grosse, 2011, Creacy et al., 2008, Gao et al., 2019, Lee and Pelletier, 2016, McRae et al., 2017, Ribeiro de Almeida et al., 2018, Thandapani et al., 2015). Therefore, expansion of our current knowledge of RG4-unwinding helicases as well as their molecular mechanisms and functions in all types of RNAs is in high demand. Herein, we identified both MOV10L1 and MOV10 as RG4-unwinding helicases.

A previous *in vivo* study obtained from *Mov10l1* mutant mice shows that loss of MOV10L1 helicase activities abolishes piRNA biogenesis accompanied by a remarkable accumulation of primary piRNA precursors and a failed production of MILI-bound PPIFs and enrichment of RG4 elements in piRNA precursors (Vourekas et al., 2015, Zheng and Wang, 2012). An initial “translocation and stalling” hypothesis model of MOV10L1 in piRNA biogenesis, derived predominantly from bioinformatics analyses, hypothesizes that MOV10L1 translocates the primary piRNA precursor transcript in a 5′ to 3′ direction feeding the precursor to the endonuclease and that it facilitates cleavage of the transcript into PPIFs at the time the helicase stalls to resolve RNA secondary structures (Vourekas et al., 2015). This model would be more specific if details about MOV10L1-RG4 interaction could be incorporated, which is essential for a thorough understanding of how PPIFs are generated, because RG4 may be an obstacle against the occurrence of one cutting on PPIFs or the movement of MOV10L1 forward. The current voids in understanding the piRNA pathway stem from poorly defined molecular events at the biochemical level. In this study, we comprehensively characterized the activity of the MOV10L1 helicase and confirmed that MOV10L1-mediated RG4 unwinding can be operated in a 5′ to 3′ directional manner when either ssRNA trap or endonuclease was present. The RNA structure-specific binding and unwinding of MOV10L1 necessitates both the N and C termini. Only after RG4 is resolved by MOV10L1, endonuclease begins to cleave the RG4 transcripts. Based on our biochemical data presented herein, we update the initial model of MOV10L1-mediated piRNA biogenesis (Figure 6C): MOV10L1 prefers to directly bind a primary piRNA precursor transcript at the junction between the ssRNA and RG4; the association of the endonuclease may result in promoting the resolution of RG4 and presumably a cutting thereon, generating PPIFs that are bound by PIWI proteins; once the local RG4 structure is completely resolved, MOV10L1 has to leave for targeting a next RG4 locus because of its low affinity to ssRNA. We deem that the resolution of RG4 is intimately coupled with the endonucleolytic cleavage for two reasons: first, RG4 resolution is required for the endonucleolytic cleavage, and second, the endonucleolytic cleavage may prevent spontaneous reformation of the RG4 after it is resolved by MOV10L1. Here, an instance obtained from integrative bioinformatics analyses was illustrated to show the *in vivo* tight correlation between MOV10L1 footprints and MILI-bound PPIFs and RG4 sites, and importantly, the necessity of RG4 resolution to form all PPIFs (Figure S8). A recent study proposes a unified piRNA biogenesis model in which PIWI guides the stepwise, phased fragmentation of a full primary piRNA precursor (Gainetdinov et al., 2018), prompting us to reason that MOV10L1 may be important for clearing up structural obstacles in the piRNA precursor for PIWI to complete the phasing process.

Although the genetic information regarding the piRNA pathway is ample, how primary piRNA precursor transcripts are distinguished from many other RNA transcripts to enter the piRNA biogenesis pathway is obscure (Le Thomas et al., 2014). Previous studies propose the mechanisms by which piRNA precursors are processed in mammalian testis (Vourekas et al., 2012, Vourekas et al., 2015), yet it remains obscure how piRNA transcripts are selected for the downstream processing. It is well recognized that RNA sequence and structural features and RNA-binding proteins play a combined role in determining RNA fates. In addition, emerging evidence now supports the notion that the functions of helicases are much broader than catalyzing strand separation (McGlynn, 2013, Sun and Wang, 2016), and that they often display structure-specific binding affinity and this specificity is postulated to confer their exact mechanisms and functions (Anand and Khan, 2004, Popuri et al., 2008). Our present data showed that MOV10L1 is a helicase with a stronger RG4-unwinding capability and a more specific RG4 binding affinity compared with MOV10 (Figures 2 and 3). In detail, MOV10L1 binds more strongly, if not specifically, to 5′-tailed RG4 structures; MOV10L1 binds poorly or even not at all to ssRNA, at least shown from the standard EMSA assay (Figure 3). Previous studies showed that MOV10L1 can associate with ssRNA when they are cross-linked (Vourekas et al., 2015). The discrepancy between the EMSA and the cross-linking result could be explained by the capacity of the latter to detect more transient or less stable interactions. Hence, an alternative explanation is that MOV10L1 quickly dissociates or translocates off the ssRNA and this is not captured by EMSA, unless encountering structures like RG4s. Thus, these results create a scenario that the pronounced RG4s (perhaps along with other secondary structures) in piRNA precursors, relatively to other transcripts, could be competitively and stably captured by MOV10L1 whereby to dictate which transcripts enter intermitochondrial cement (IMC)/nuage before undergoing the subsequent piRNA processing. Interestingly, MOV10L1's fly ortholog Armi has already been proved to be an essential factor in identifying a piRNA precursor by tethering to the transcript (Pandey et al., 2017). Tudor domain protein TDRD5 functions in selective processing of the piRNA cluster transcripts after being recruited to IMC (Ding et al., 2018). It is conceivable that the initial recruitment of piRNA precursors that distinguishes between transcripts marked as piRNA and non-piRNA may require other elements or proteins.

Effective nucleic acid binding and unwinding of helicase often ask for the functional interplay between the helicase core and ancillary domains as well as auxiliary partners (Fiorini et al., 2013, Rudolph and Klostermeier, 2015). This notion is also valid for the MOV10L1 helicase. The requirements of both the N- and C termini of MOV10L1 in RNA binding and unwinding is in stark contrast to one of its closest homologs UPF1 in which both the N- and C-terminal domains inhibit HD domain in unwinding (Fiorini et al., 2013). Its activation requires the additional partners UPF2 and UPF3 (Chakrabarti et al., 2011, Lykke-Andersen et al., 2000, Lykke-Andersen and Jensen, 2015). This auto-inhibitory mechanism of human UPF1 reflects its intricate enzymatic control and regulation of its helicase activity. MOV10L1 seems not to use flanking domains but instead employs auxiliary partners in regulation of its helicase activity. As evident here, it alone can hardly resolve RG4 even if both termini are present (Figure 5). We report evidence suggesting RG4 cleavage by an endonuclease is facilitated by the presence of MOV10L1 (Figure 6). This feature ensures the loading of required proteins before proceeding. Similarly, this stimulation was also observed with a recent reported RG4-unwinding helicase DDX21 (McRae et al., 2017). It is likely that RNA helicase slips during unwinding as previously reported for other helicases (Lee et al., 2014, Sun et al., 2011), and timely cleavage of unwound RG4 by endonuclease prevents them from backward movement, thus ensuring continuous RG4 unwinding. Based on the overall limited sequence homology between MOV10L1 and MOV10, our study also offers clues to how paralogs of the mammalian helicase may evolve their biochemical discrepancy in correspondence to their distinct biological implication.

### Limitation of the Study

Although we provided evidence that in our experimental conditions the unwinding activities per microgram for each protein can be considered being comparable, a further more detailed characterization would be required to demonstrate that the recombinant proteins are folded correctly, uniformly, and that they show the same activity per microgram of protein mass.

## Methods

All methods can be found in the accompanying Transparent Methods supplemental file.

## Supplemental Information

Document S1. Transparent Methods, Figures S1–S8, and Tables S1 and S2
