# Supplementary material for: MOV10L1 Binds RNA G-Quadruplex in a Structure-Specific Manner and Resolves It More Efficiently Than MOV10
Source: iScience. 2019 Jun 15;17:36–48. doi: 10.1016/j.isci.2019.06.016 (PMC6600044; doi:10.1016/j.isci.2019.06.016)
Supplement: Document S1. Transparent Methods, Figures S1–S8, and Tables S1 and S2 [file mmc1.pdf]

ISCI, Volume *17*

Supplemental Information

MOV10L1 Binds RNA G-Quadruplex in a Structure-Specific Manner and Resolves It More Efficiently Than MOV10

Xia Zhang, Lina Yu, Shasha Ye, Jie Xie, Xingxu Huang, Ke Zheng, and Bo Sun

Table S1. Sequences of RNA/DNA substrates used in this work. Related to Figures 1-6.

For figures	Names	Sequences (From 5' to 3')
Figure 1, 2, 3, 5	5'- tailed RG4	5'- UUUUUUACCGGUGGUG**GGG**A**GGG**UCC**GGG**U**GGG**A /cy3/
Figure 1, 2, 5, S4	RG4 trap	5'- GGGCCCUCCCCGCUUGCCCGGUCGU
Figure 2, 3	RG4 only	5'- G**GGG**A**GGG**UCC**GGG**U**GGG**A/cy3/
Figure 3	18 nt ssRNA	5'- /cy3/ACCGCUGCCGUCGCUCCG
Figure 4	5'- tailed DG4	5'- TTTTTTACCGGTGGTG**GGG**A**GGG**TCC**GGG**T**GGG**A /cy3/
	DG4 trap	5'- GGGCCCTCCCCGCTTGCCCGGTCGT
	D/RNA	5'- /cy3/ ACCGCTGCCGTCGCTCCG<br>5'- ACGAGGGAGACGAGGAGACGGAGCGACGGCAGCGGU
	R/DNA	5'- /cy3/ ACCGCUGCCGUCGCUCCG<br>5'- ACGAGGGAGACGAGGAGACGGAGCGACGGCAGCGGT
	dsDNA	5'- /cy3/ ACCGCTGCCGTCGCTCCG<br>5'- ACGAGGGAGACGAGGAGACGGAGCGACGGCAGCGGT
	DNA trap	5'- ACCGCTGCCGTCGCTCCG
Figure 4, S2, S3, S5, S6	5'- tailed duplex	5'- /cy3/ ACCGCUGCCGUCGCUCCG<br>5'- ACGAGGGAGACGAGGAGACGGAGCGACGGCAGCGGU
	RNA trap	5'- ACCGCUGCCGUCGCUCCG
Figure S5	Forked duplex	5'- /cy3/ ACCGCUGCCGUCGCUCCGAGAGAGAGCA<br>5'- ACGAGGGAGACGAGGAGACGGAGCGACGGCAGCGGU
	Blunt duplex	5'- /cy3/ ACCGCUGCCGUCGCUCCG<br>5'- CGGAGCGACGGCAGCGGU
Figure 6，S7	5'- tailed RG4-2	5'- UUUUUUACUAUCACUU**GGG**A**GGG**U**GGG**C**GGG**UA /cy3/
Figure S7	ssRNA trap-2	5'- ACCCUCCCAAGUGAUAGUCGU

RNA and DNA sequences are colored in red and black respectively. The guanines that form G-quadruplex are highlighted in bold. The complementary sequences in RG4 (DG4) substrate and traps are marked with points below.

Table S2. The primers used to construct plasmids. Related to Figure 5.

Mutants	Primers	Sequences (From 5' to 3')
MOV10L1 - HD	forward	5’-GACGACGATGACAAG**GGATCC**ATGATCCTTTTTGGACCTCCGGGA -3’
	reverse	5’-GCTTACTCAGCTAAG**CTCGAG**TCAAGGGTTTCCCAGAATGATCAGC -3’
MOV10L1 - ΔN	forward	5’-GACGACGATGACAAG**GGATCC**ATGATCCTTTTTG GACCTCCGGGA-3’
	reverse	5’-GCTTACTCAGCTAAG**CTCGAG**TCACTTTTGGAGAG CCTG-3’
MOV10L1 - ΔC	forward	5’-GACGACGATGACAAG**GGATCC**ATGCTGCGCCTT GCTGCCAA -3’
	reverse	5’- GCTTACTCAGCTAAG**CTCGAG**TCAAGGGTTTCCCAGA ATGA -3’
MOV10	forward	5’-GACGACGATGACAAG**GGATCC**ATGCCTAGCAAGTTCAGCTGCCGAA-3’
	reverse	5’- GCTTACTCAGCTAAG**CTCGAG**TCAGAGCTCATTTCTCCACTCTGGC -3’

The highlighted sequences in forward and reverse primers are the BamH I site and the Xho I site, respectively.

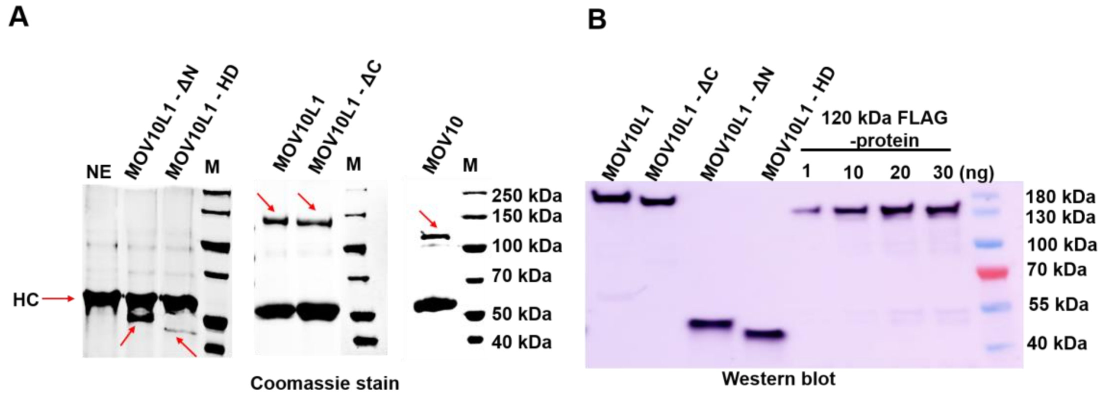

Figure S1. Purification of MOV10L1 and MOV10 proteins. Related to Figures 1-6.

(**A**) Coomassie stain of FLAG purified wild type MOV10L1, MOV10L1 mutants and MOV10 proteins. 50 μl of purified proteins-bound beads were heated at 95°C for 10 min, and then resolved on 8% SDS-PAGE gel and then stained by Coomassie R250. The red arrows indicated the positions of the purified proteins. Bands on the coomassie gel with a molecular weight of approximately 55 kDa correspond to the heavy immunoglobulin chain (IgG) from the FLAG antibody and the antibody accumulation. NE denotes no enzyme.

(**B**) Western blot analyses of FLAG purified wild type MOV10L1 and truncated mutants. The molecular weights of proteins are 137 kDa (MOV10L1), 128 kDa (MOV10L1 - ΔC), 46.5 kDa (MOV10L1 - ΔN) and 43 kDa (MOV10L1 - HD). The MOV10L1 proteins and a 120 kDa FLAG-protein of known concentration were resolved by 8% SDS-PAGE gel. The gel was transferred to a PVDF membrane. Membrane was blocked in TBS containing 0.1% Tween-20 (TBS-T) and 5% skimmed milk powder. Anti-FLAG (sigma) antibody was diluted as 1: 5000 and incubated with the membrane for 1 h at room temperature in TBS-T with 5% milk. Membrane was washed 3 times in TBS-T and then incubated with the secondary antibody (1:10000 diluted) conjugated to horse radish peroxidise (HRP) for 0.5 h at room temperature in TBS-T with 5% milk. Membrane was washed 3 times in TBS-T and then proteins were visualized on Amersham Imager 600 (GE Healthcare) by enhanced chemiluminescence (Bio-Rad). The concentrations of the MOV10L1 proteins were determined by comparing with the FLAG-protein of known concentration on the gel.

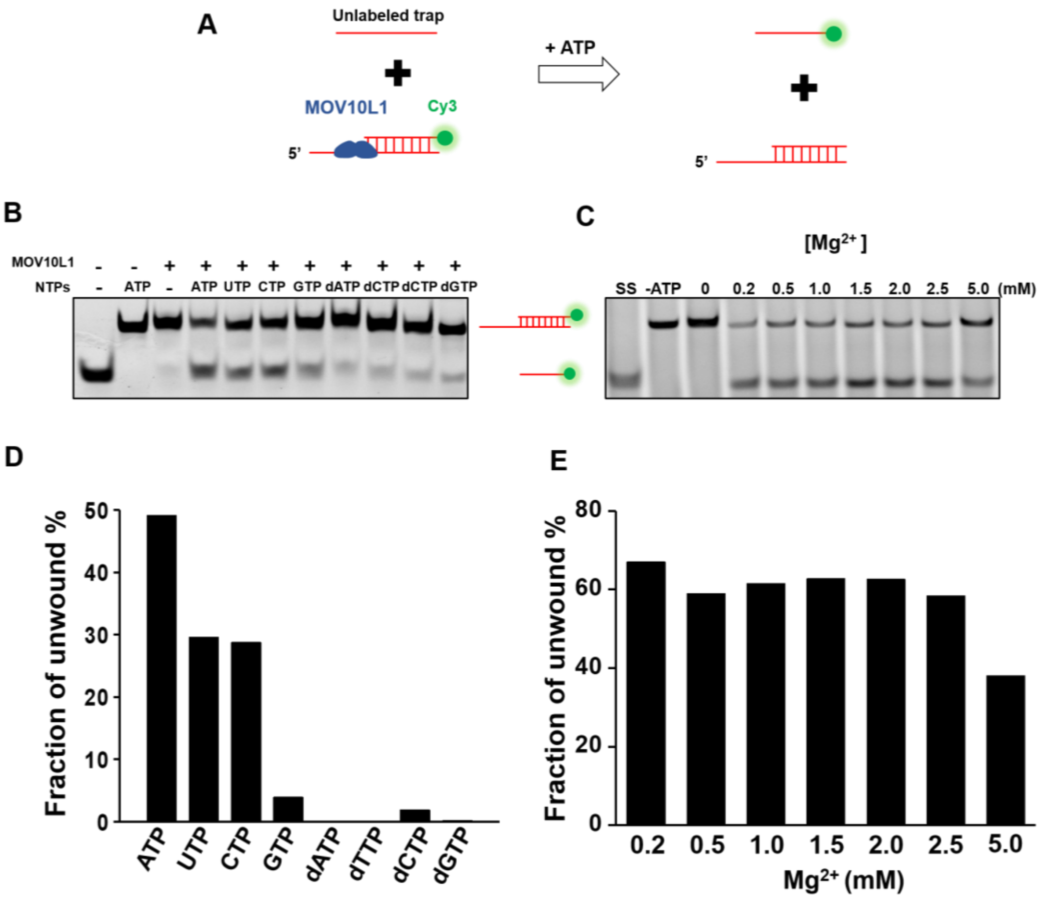

Figure S2. The optimal condition for MOV10L1 unwinding 5'-tailed RNA duplex. Related to Figure 1.

(**A**) Schematic demonstrating the principle of the RNA-duplex unwinding assay. RNA duplex substrate was generated by annealing a cy3-labeled oligo with its complementary strand. An excess amount of an unlabeled ssRNA with the same sequence as the labeled strand was added in the reaction to prevent reannealing. The *in vitro* unwinding reactions were carried out by pre-incubating MOV10L1 with the substrate in the presence of Mg^{2+}, followed by initiating the reactions with various NTPs and a displaced strand oligonucleotide trap.

(**B-C**) A representative image of MOV10L1-mediated 5'-tailed dsRNA unwinding reactions for 180 min at 37°C with different nucleoside triphosphates or different concentrations of Mg^{2+}. This experiment was repeated three times.

(**D-E**) The quantitative analyses of gels in panels *B* and *C*.

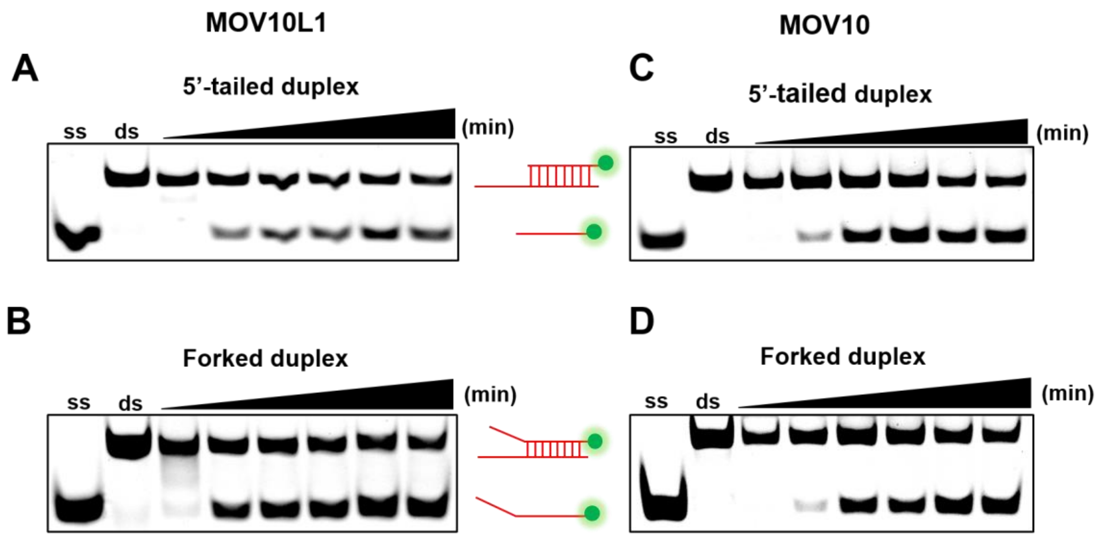

Figure S3. **MOV10L1 unwinds 5'- tailed and forked RNA substrates. Related to Figure 2.**

Representative gels of MOV10L1- and MOV10-mediated unwinding of 5'- tailed duplex (**A** and **C**) and forked duplex (**B** and **D**) with increasing time (0, 10, 30, 60, 120, 180 min) at 37°C in the presence of 10-fold of the ssRNA trap. The quantification analyses were shown in Figure 2C and 2D.

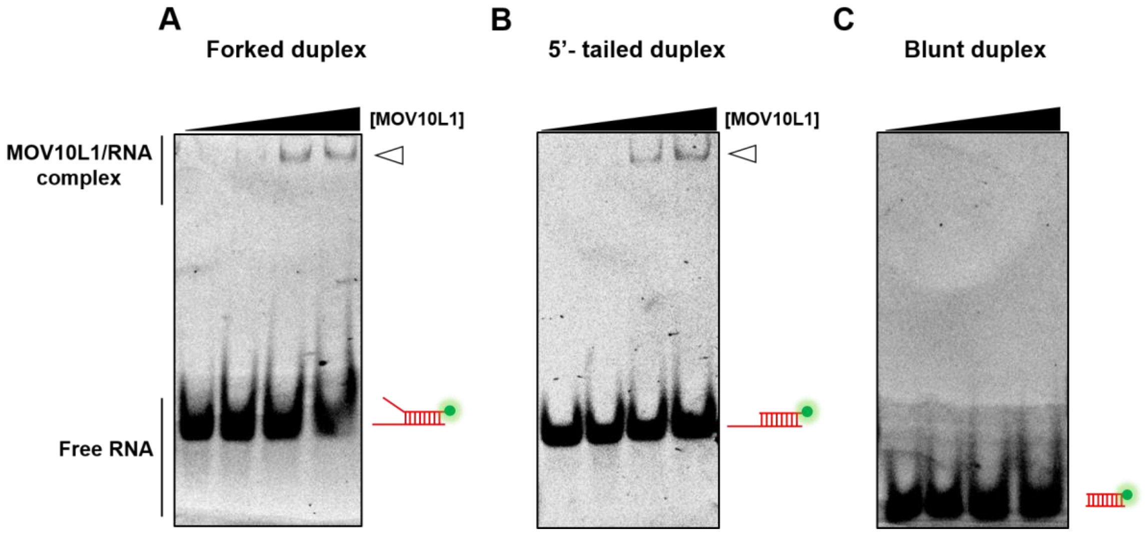

Figure S4. MOV10L1 binds to RNA duplex substrates bearing an ssRNA tail. Related to Figure 3.

Representative gels of increasing amounts of MOV10L1 proteins (0, 2, 10, 20 ng) binding to forked duplex (**A**), 5' tailed duplex (**B**) and blunt duplex (**C**). The experimental procedures were described in the Transparent Methods section and this experiment was repeated three times. MOV10L1/RNA complexes were detected in the presence of the forked duplex and the 5'-tailed duplex substrate but not in the presence of the blunt duplex substrate. These results confirmed that the MOV10L1 helicase preferentially binds to a junction formed by ssRNA and structured RNA.

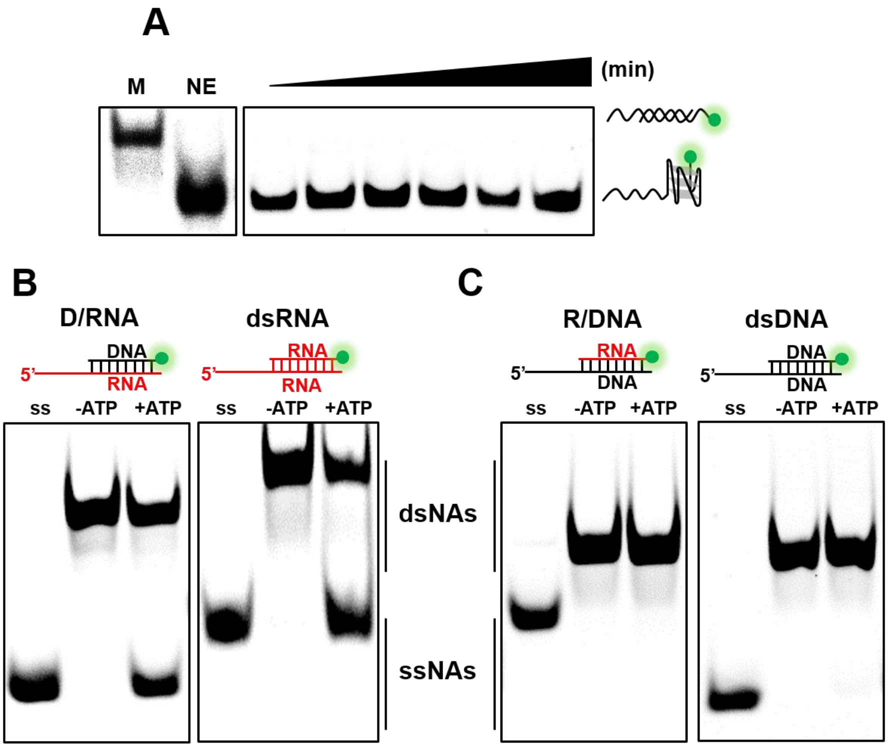

Figure S5. MOV10 is unable to unwind DG4. Related to Figure 4.

(**A**) A representative gel of MOV10-mediated unwinding of DG4 with increasing time (0, 10, 30, 60, 120, 180 min) at 37°C in the presence of 10-fold of the ssRNA trap. Marker (denoted as M) was prepared as described in the Materials and Methods section. NE denotes no enzyme.

(**B, C**) MOV10 unwound different duplex substrates (D/RNA, dsRNA, R/DNA and dsDNA, 10 nM each) with or without ATP at 37°C for 180 min. dsNAs and ssNAs denote double-stranded nucleic acids and single-stranded nucleic strands respectively. The quantitative analyses of these gels were shown in Figure 4D.

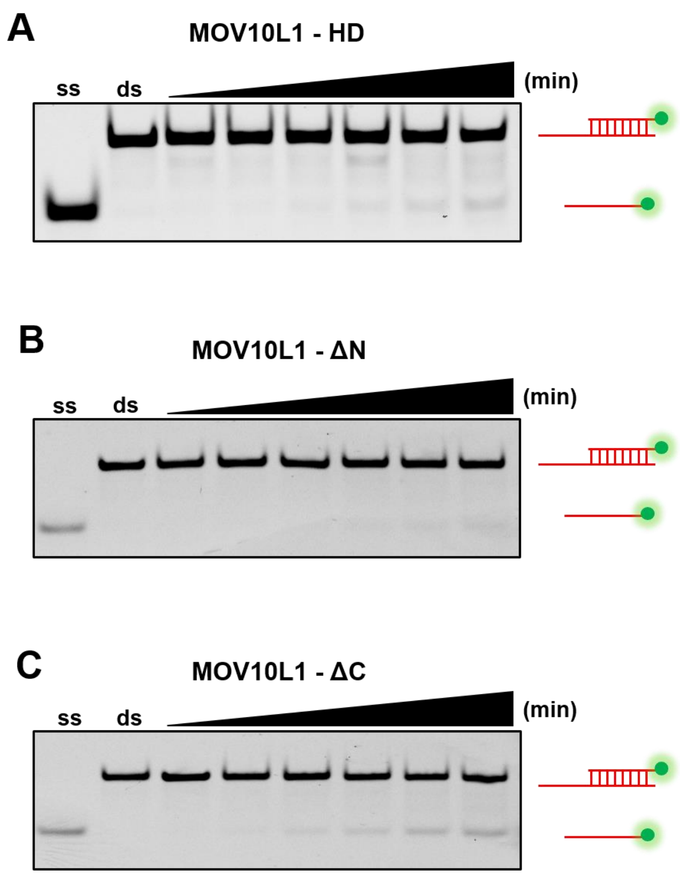

Figure S6. Both the N- and C- termini of MOV10L1 are required for dsRNA unwinding. Related to Figure 5.

Representative gels of MOV10L1 mutants, MOV10L1 - HD (**A**), MOV10L1 - ΔN (**B**) and MOV10L1 - ΔC (**C**), unwinding 5'-tailed RNA duplex with increasing time (0, 10, 30, 60, 120 and 180 min) at 37°C. This experiment was repeated three times. The experimental procedure of this RNA duplex-unwinding assay was described in the Transparent Methods section. All the mutants exhibited little to none unwinding activities on this substrate and these results further confirmed that both the N- and C- termini of MOV10L1 are required for its efficient RNA unwinding.

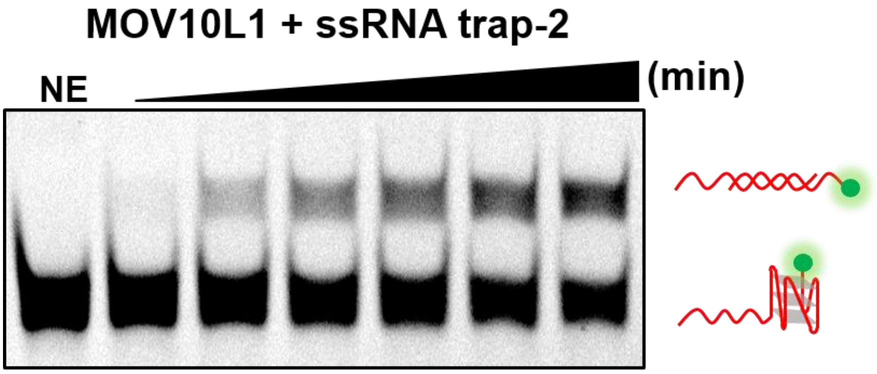

Figure S7. MOV10L1 unwinds RG4-2 in the presence of the ssRNA trap. Related to Figure 6.

A representative gel of MOV10L1 unwinding 5'-tailed RG4-2 in the presence of the ssRNA trap-2 with increasing time (0, 2, 5, 10, 20 and 30 min) at 37°C. The experimental procedures were described in the Transparent Methods section and this experiment was repeated three times. In the presence of the ssRNA trap-2, RG4 unwinding occurred and the unwinding products increased over time.

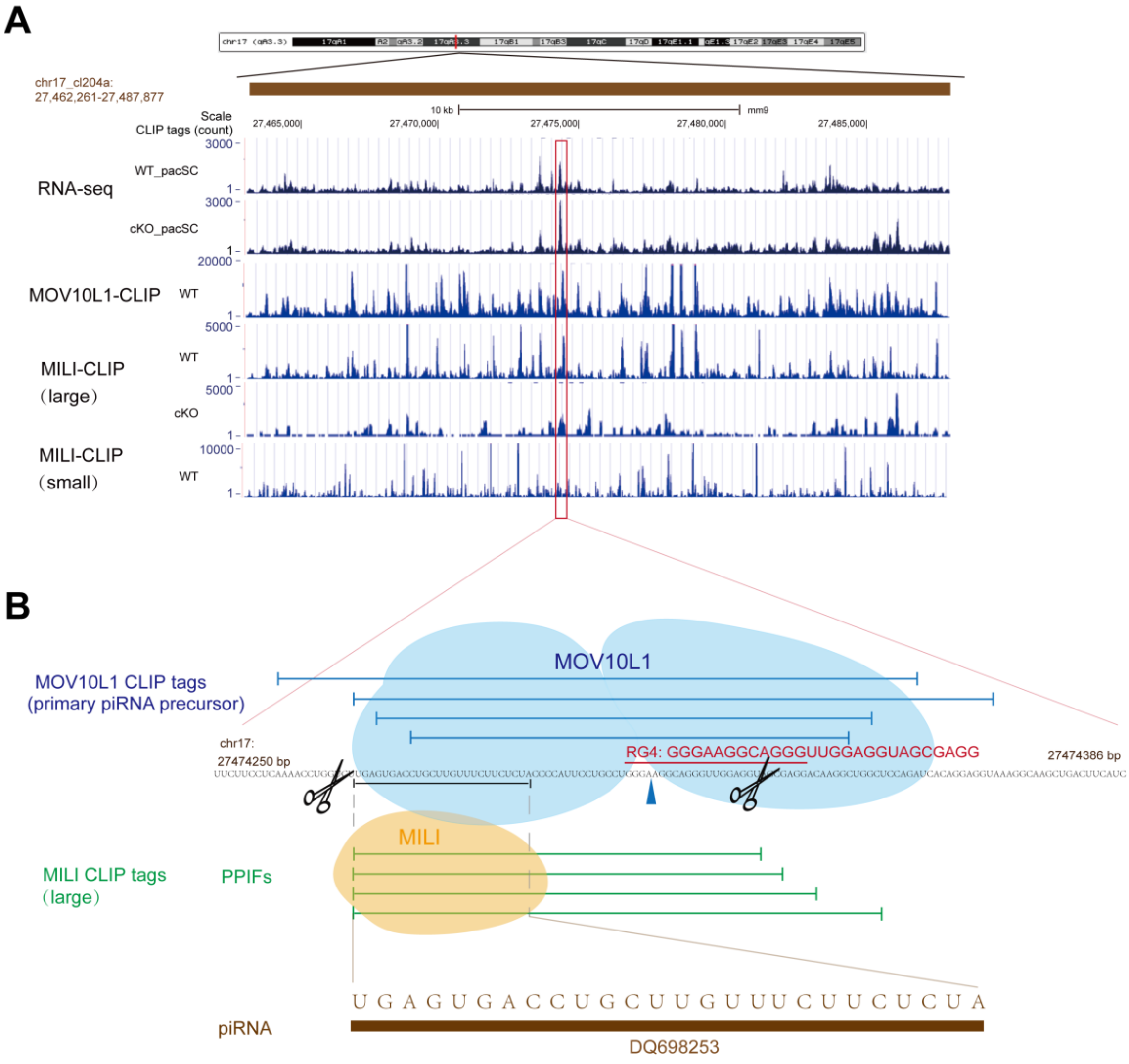

Figure S8. Bioinformatics illustration of the primary piRNA processing mediated by MOV10L1-RG4 interaction. Related to Figure 6.

(**A**) UCSC genome browser snapshot of pacSC RNA-seq reads, MOV10L1 CLIP tags, MILI CLIP tags (small: piRNAs; large: PPIFs) mapping within the pachytene piRNA cluster (chr17_cl204a: 27,462,261-27,487,877). One typical area occupied by peaks from the RNA-seq, MOV10L1 CLIP, and MILI CLIP (large) libraries which show visibly lower PPIFs counts in *Mov10l1*^{cKO} is marked with a red box. All deep-sequencing data above mapping to the mouse genome (mm9) are requested from two previous reports (Vourekas et al., 2015; Vourekas et al., 2012), and shown here as normalized counts.

(**B**) A magnified view of representative sequences located within the typical area indicated in panel A shows that the locations of primary piRNA precursor, RG4, MOV10L1 footprints and MILI-bound PPIFs as well as its corresponding piRNA are interdependent. Based on our biochemical results,

MOV10L1 prefers to directly bind in vicinity to the approximate midpoint (blue arrowhead) of its CLIP tags (blue lines aligned to the primary piRNA precursor), adjacently downstream of which is the area of RG4 structure (a red lined sequence conforming to RG4 prediction algorithms). In this case, two cleavages (one scissor ahead of and the other right on RG4) might occur before and after MOV10L1 starts to resolve RG4, respectively. The fragments cut out from the primary piRNA precursor are bound by MILI, yielding 5'-end-stabilized PPIFs (green lines) which are further trimmed at their 3'-end to form the mature piRNA (brown lined sequence).

Transparent Methods

Nucleic acid substrates

HPLC-purified RNA and DNA oligonucleotides with or without labels used to make the substrates or traps were purchased from TaKaRa (Dalian, China) and Sangon Biotech (Shanghai, China), respectively (Table S1). Each oligo was diluted to 20 μM in RNase free water and kept at -80°C for further use. Duplex DNA or RNA substrates were generated by heating a mixture of a cy3-labeled oligo (2 μM, final concentration) with a 1.2-fold of its unlabeled complementary strand in the annealing buffer (60 mM HEPES pH 7.5, 6 mM KCl, 0.2 mM $MgCl_2$) at 95°C for 5 min followed by slow cooling to room temperature. RNA or DNA G4 substrates were prepared by heating an ssDNA or ssRNA oligo in the G4 formation buffer (20 mM Tris-HCl pH 7.5, 100 mM KCl, 1 mM EDTA) at 95°C for 5 min followed by slow cooling to room temperature.

Circular dichroism (CD) spectropolarimetry

After forming the RG4, a 1,200 μl sample of this substrate at the final concentration of 1.5 μM in buffer containing 20 mM Tris-HCl, pH 7.5 and 20 mM KAc was used for the CD experiments which were performed on Bio-Logic MOS450/AF-CD optical system (BioLogic Science Instruments, France) using 2 mm path length quartz cells. Spectrum was recorded from 220 nm to 320 nm.

Expression and purification of MOV10L1 and MOV10 proteins

Full-length MOV10L1 was purified as described previously (Fu et al., 2016; Vourekas et al., 2015). The MOV10L1 protein was transiently expressed in HEK293T cells with TurboFect transfection reagent (Thermo). Cells were split 24 h post-transfection and then harvested next day. Cells were then lysed with CelLytic™ M Cell Lysis Reagent (sigma) and the lysates were centrifuged at 16,000 g for 30 min. The supernatants were filtered through a 0.45 μm filter to remove any remaining cell

debris and particulates. The cleared cell lysates were then mixed with 1 ml of anti-FLAG M2 magnetic beads suspension (Sigma), prewashed with K150 buffer (50 mM HEPES at pH 7.5, 150 mM KoAc, 1 mM DTT, 0.1% Igepal [NP-40] (sigma) with EDTA free protease inhibitors cocktail (Roche)), and incubated for 2 h at 4°C on rotation. The protein bound beads were washed three times with K150 buffer, twice with K150 containing 250 mM NaCl, and three more times with K150 buffer. The beads were resuspended in 1 ml of 50% glycerol in K150 buffer and then stored at -20°C for helicase unwinding assays. (Figure S1A). For MOV10L1 binding assays, the protein was eluted using 500 μl of K150 buffer containing 0.2 mg/ml 3X FLAG peptide and 10% glycerol at 4°C for 2 h on rotation. The eluted proteins were kept at -80°C for further use. The MOV10 protein and MOV10L1 mutants (K778A, DE888AA, MOV10L1-ΔC, MOV10L1-ΔN, and MOV10L1-HD) were expressed and purified using the same protocol. Protein concentrations were determined by western blot analyses (Figure S1B). 10 μl of MOV10L1/MOV10 -bound bead suspension or eluted solution carried about 20 ng of protein. All primers used to construct the plasmids were listed in Table S2.

Helicase unwinding assays

A previously developed RG4-unwinding assay was adopted to detect MOV10L1's G4 unwinding activity with modifications (Booy et al., 2015; Booy et al., 2012). Briefly, MOV10L1-bound bead suspension was washed three times in reaction buffer (50 mM Tris-HCl pH 7.5, 20 mM KoAc, 2 mM $MgCl_2$, 0.01% Igepal [NP-40], 1 mM DTT) before unwinding assays. 10 μl of the suspension (20 ng of MOV10L1) was used for each assay unless indicated otherwise. The bead suspension was mixed with RG4 substrate (10 nM, final concentration) in the reaction buffer with 2U/μl RNase inhibitor. The mixture was incubated at 37°C for 10 min with shaking in a thermomixer (1000 rpm). Then, 2 mM ATP and 100 nM RNA or DNA traps were added to the mixture (20 μl, final volume) to initiate the unwinding reaction. The reactions were incubated at 37°C for indicated times with shaking and

stopped by the addition of 5X stop buffer (125 mM EDTA, 50% glycerol). Lastly, protease K (TIAN GEN) was added to a finial concentration of 2 μg/μl for another 10 min at 37°C to degrade the protein. Reaction products were resolved on a 12% native TBE PAGE gel at 120 V for 40 min. To illustrate the expected mobility of the unfolded RG4 annealed with trap, 100 mM LiCl was substituted for 100 mM KCl in the G4 formation buffer with equal amount of RG4 trap (Booy et al., 2012). For the DG4 substrate, a heat denatured DG4 substrate was annealed to a 250-fold of DG4 trap in the G4 formation buffer to illustrate the unfolded DG4 on the gel (Giri et al., 2011; Wu and Spies, 2016).

We used a previously described RNA duplex-unwinding assay to detect the duplex-unwinding activity of MOV10L1 and MOV10 with some modifications (Jankowsky and Putnam, 2010; Vourekas et al., 2015). This assay was similar to the RG4-unwinding assay except that the unwinding products were indicated by ssRNA instead of dsRNA on the TBE PAGE gel. All the experiments were carried out at least three times and representative gels and plots were shown.

Electrophoretic Mobility Shift Assay (EMSA)

To form RNA/protein complexes, we incubated the indicated concentrations of purified proteins with 2.5 nM of a cy3-labeled RNA substrate in binding buffer (50 mM Tris-HCl pH 7.5, 100 mM KoAc, 2 mM $MgCl_2$, 2 mM AMP-PNP or ATP, 2% glycerol, 2 U of RiboLock RNase Inhibitor, 1 mM DTT) at 37°C for 30 min. After the incubation, 5X stop buffer was added to each mixture, and samples were resolved on 8% native TBE PAGE gel at 90 V for 1 h at room temperature. All the experiments were carried out at least three times and representative gels and plots were shown.

ATPase assay

ATPase activities of all tested proteins were measured using an ATPase activity assay kit (MAK113, Sigma). Briefly, recombinant MOV10L1 proteins bound beads were prewashed three times by the

ATPase assay buffer (50 mM Tris-HCl pH 7.5, 20 mM KoAc, 0.2 mM $MgCl_2$, 1 mM DTT), followed by incubation with 0.2 mM ATP in the ATPase buffer and 5 nM 5'-tailed RG4. Reactions were allowed to proceed at 37°C for 60 min or 120 min. The reactions were stopped by addition of 200 μl reagent MAK113A in each reaction and incubated 5 min at room temperature. The absorbance at 620 nm was then measured using the SpectraMax i3x (Molecular devices) for all samples. All the experiments were carried out at three times.

RNase T1 digestion

The experimental procedures were same as the RG4 unwinding assay except that the ssRNA trap was replaced by RNase T1 (50 U/μl, final concentration) (Thermo). The samples were thoroughly mixed and incubated at 37°C for 0, 2, 5, 10, 20 and 30 min. The reactions were stopped by the addition of 5X stop buffer, followed by being resolved on 5% native TBE PAGE gel. This experiment was carried out three times and representative gels and plots were shown.

Gel imaging and quantification

Unwinding and binding products were detected by Phosphor-Imaging, scanned on a Typhoon FLA 9500 Imager (GE Healthcare) and analyzed with Image Quant TL software (Nonlinear Dynamics). Enzymatic unwinding was calculated after subtraction of the fraction of non-enzymatically dissociated substrate observed in control reactions. The fraction of unwound RNA against time was fit to the pseudo-first order equation, $F=A*(1-e^{-kt})$, where A is the maximum fraction of unwound/cleaved RNA that can be generated enzymatically from the substrates; k is the rate constant of RNA unwinding/cleavage; t is reaction time. For comparison, the unwinding rates in Figure 4D were calculated from the formula (unwound substrate/total substrate) x nM substrate/μM enzyme/time.

Supplemental References

Booy, E.P., McRae, E.K., and McKenna, S.A. (2015). Biochemical characterization of G4 quadruplex telomerase RNA unwinding by the RNA helicase RHAU. Methods Mol Biol *1259*, 125-135.

Booy, E.P., Meier, M., Okun, N., Novakowski, S.K., Xiong, S., Stetefeld, J., and McKenna, S.A. (2012). The RNA helicase RHAU (DHX36) unwinds a G4-quadruplex in human telomerase RNA and promotes the formation of the P1 helix template boundary. Nucleic Acids Res *40*, 4110-4124.

Fu, Q., Pandey, R.R., Leu, N.A., Pillai, R.S., and Wang, P.J. (2016). Mutations in the MOV10L1 ATP Hydrolysis Motif Cause piRNA Biogenesis Failure and Male Sterility in Mice. Biol Reprod *95*, 103.

Giri, B., Smaldino, P.J., Thys, R.G., Creacy, S.D., Routh, E.D., Hantgan, R.R., Lattmann, S., Nagamine, Y., Akman, S.A., and Vaughn, J.P. (2011). G4 resolvase 1 tightly binds and unwinds unimolecular G4-DNA. Nucleic Acids Res *39*, 7161-7178.

Jankowsky, E., and Putnam, A. (2010). Duplex unwinding with DEAD-box proteins. Methods Mol Biol *587*, 245-264.

Vourekas, A., Zheng, K., Fu, Q., Maragkakis, M., Alexiou, P., Ma, J., Pillai, R.S., Mourelatos, Z., and Wang, P.J. (2015). The RNA helicase MOV10L1 binds piRNA precursors to initiate piRNA processing. Genes Dev *29*, 617-629.

Vourekas, A., Zheng, Q., Alexiou, P., Maragkakis, M., Kirino, Y., Gregory, B.D., and Mourelatos, Z. (2012). Mili and Miwi target RNA repertoire reveals piRNA biogenesis and function of Miwi in spermiogenesis. Nat Struct Mol Biol *19*, 773-781.

Wu, C.G., and Spies, M. (2016). G-quadruplex recognition and remodeling by the FANCJ helicase. Nucleic Acids Res *44*, 8742-8753.
